# Effect of spirometry on intra-thoracic pressures

**DOI:** 10.1186/s13104-018-3217-9

**Published:** 2018-02-08

**Authors:** Nicholas B. Tiller, Andrew J. Simpson

**Affiliations:** 1 Academy of Sport and Physical Activity, Sheffield Hallam University, Sheffield, S10 2BP UK; 2 Centre for Human Performance, Exercise and Rehabilitation, Brunel University, London, UK; 3 Department of Sport, Health and Exercise Science, University of Hull, Hull, UK

**Keywords:** Spirometry, Pressure, intra-thoracic pressure, Pulmonary function, Lung function, Balloon catheter

## Abstract

**Objective:**

Due to the high intra-thoracic pressures associated with forced vital capacity manoeuvres, spirometry is contraindicated for vulnerable patients. However, the typical pressure response to spirometry has not been reported. Eight healthy, recreationally-active men performed spirometry while oesophageal pressure was recorded using a latex balloon-tipped catheter.

**Results:**

Peak oesophageal pressure during inspiration was − 47 ± 9 cmH_2_O (37 ± 10% of maximal inspiratory pressure), while peak oesophageal pressure during forced expiration was 102 ± 34 cmH_2_O (75 ± 17% of maximal expiratory pressure). The deleterious consequences of spirometry might be associated with intra-thoracic pressures that approach maximal values during forced expiration.

## Introduction

Spirometry is the most common pulmonary function test for the diagnosis and monitoring of respiratory disorders. A forced vital capacity (FVC) manoeuvre is initiated via the co-contraction of several inspiratory muscles including the diaphragm, external intercostals, and the accessory inspiratory muscles (scalenes and sternocleidomastoids), causing a sharp fall in intra-thoracic pressure, and subsequent inspiratory airflow. Following the attainment of total lung capacity (TLC), the patient rapidly contracts the major expiratory muscles (e.g., rectus abdominis, internal intercostals, external obliques), which generates large positive pressures in the thorax, and a subsequent maximal forced expiration to residual volume (RV). In healthy participants, spirometry is considered both safe and reproducible [1].

Spirometry is, however, contraindicated for vulnerable populations including patients with recent cardiac complications or those having recently undergone major surgery [1]. Moreover, spirometry is associated with bronchoconstriction [2], cardiac arrhythmia [3], and gastro-oesophageal reflux [4]. The mechanisms that underpin these negative consequences are unclear, although they may relate to the large intra-thoracic pressures associated with maximal, dynamic respiratory manoeuvres. Intra-thoracic pressures during the FVC manoeuvre have not been characterised, but such data would inform our understanding of the respiratory-mechanical response to spirometry. Accordingly, we aim to report oesophageal pressure (Pes)—a common surrogate for intra-thoracic pressure—during spirometry in healthy men.

## Main text

### Methods

#### Study subjects

Eight healthy, non-smoking, recreationally-active men volunteered to participate (mean ± SD: age 24 ± 5 years; stature 1.79 ± 0.07 m; mass 74 ± 11 kg). Subjects completed a pre-participation health questionnaire, and were free from any known cardiorespiratory disorders. At the time of testing, subjects were physically-active, but were not engaged in any specialist athletic training. Experimental procedures were approved by the institution Research Ethics Committee, performed according to the Declaration of Helsinki, and written informed consent was provided.

#### Study design

Participants performed an FVC manoeuvre into a phlanged mouthpiece connected to a low-resistance, bidirectional turbine, with measurements recorded using an online gas analyser (Oxycon Pro #791965, Jaeger GmbH, Hoechberg, Germany). Intra-thoracic pressure was estimated via oesophageal pressure [4] measured using a balloon-tipped catheter (#47-9005-5Fr, Ackrad Labs, Cooper Surgical, Berlin, Germany) connected to a differential pressure transducer (#DP45 LPV Reluctance Sensor; Validyne range ± 229 cmH2O), which was calibrated across the physiological range. The catheter was inserted pernasally into the stomach, filled with 1 mL of air, and withdrawn until the diaphragm produced a negative pressure deflection on inspiration. The balloon was then withdrawn a further 10 cm so that the distal end was situated in the lower one-third of the oesophagus. Oesophageal pressures during both the inspiratory (P_es,insp_) and expiratory (P_es,exp_) portions of the FVC manoeuvre were expressed in absolute terms and as a percentage of the maximal static inspiratory pressure (P_Imax_) and expiratory pressure (P_Emax_) recorded from residual volume and total lung capacity, respectively. All respiratory manoeuvres were performed in accordance with recommended standards [5].

### Results

**Fig. 1:**
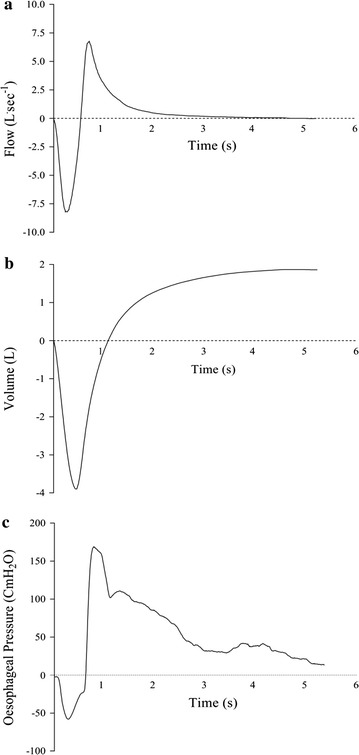
Representative flow (**a**), volume (**b**), and oesophageal pressure (**c**) traces from a single subject performing an FVC manoeuvre. Peak flow = 6.78 L s^−1^; volume = 5.84 L; peak inspiratory oesophageal Pressure = 58 cmH_2_O; peak expiratory oesophageal pressure = 169 cmH_2_O

Pulmonary function was within normal limits (see Table 1) [6]. Oesophageal pressure during the inspiratory portion of the FVC reached a peak value of − 47 ± 9 cmH_2_O, which was equivalent to 37 ± 10% P_Imax_. Oesophageal pressure during the expiratory portion of the FVC reached a peak value of 102 ± 34 cmH_2_O, which was equivalent to 75 ± 17% P_Emax_. Consequently, the oesophageal pressure swing (ΔPes) during spirometry was 149 ± 40 cmH_2_O. Representative data for flow, volume and oesophageal pressure during an FVC manoeuvre are shown in Fig. 1.

**Table 1:** Baseline (resting) pulmonary function in eight healthy, recreationally-active men

	Absolute	Relative	
FVC, L	5.71 ± 0.51	102 ± 6	%Pred.
FEV_1_, L	4.45 ± 0.47	95 ± 8	%Pred.
FEV_1_/FVC, %	78 ± 5	− 1 ± 0.6	z-score
Pes_insp_, cmH_2_O	− 47 ± 9	37 ± 10	%P_Imax_
Pes_exp_, cmH_2_O	102 ± 34	75 ± 17	%P_Emax_
ΔPes, cmH_2_O	149 ± 40	– ± –

Values are mean ± SD for eight participants. FVC, forced vital capacity; FEV_1_, forced expiratory volume in one second; Pes_insp_, peak oesophageal pressure recorded during inspiratory phase of FVC; Pes_exp_, peak oesophageal pressure recorded during expiratory phase of FVC; ΔPes, oesophageal pressure swing (peak-to-peak) recorded during FVC manoeuvre; P_Imax_, maximum static inspiratory oesophageal pressure; P_Emax_, maximum static expiratory oesophageal pressure. Predicted values and z scores for pulmonary volumes and flows are from Quanjer et al. [6].

### Discussion

The aim of this report was to characterise the intra-thoracic pressure-response to spirometry performed by healthy men. Our main findings were that an FVC manoeuvre resulted in a peak inspiratory oesophageal pressure of − 47 cmH_2_O (37% P_Imax_), and a peak expiratory oesophageal pressure of 102 cmH_2_O (75% P_Emax_). These large intra-thoracic pressures may have implications for respiratory health in vulnerable patients.

A forced vital capacity manoeuvre can be split into two distinct phases: (i) inspiration to total lung capacity; (ii) forced expiration to residual volume. The typical, healthy response to deep inspiration is bronchodilation [7]. In mild-to-moderate asthma, however, bronchodilation following a deep inspiration is inhibited, and in severe asthma, a deep inspiration may induce bronchoconstriction [8]. It has been suggested that spirometry-induced bronchoconstriction is caused, at least in part, by an increase in airway wall oedema, secondary to an increase in intra-thoracic pressure across the airway capillaries [8]. Moreover, cardiac complications, including myocardial infarction, aortic aneurysm, hypertension and angina, are among the most common contraindications for lung function testing [9], and may be caused by large changes in intra-thoracic pressures during spirometry, and a subsequently elevated blood pressure [9]. Indeed, arrhythmia during spirometry was observed in 10% of patients referred for cardio-pulmonary exercise testing; notably the authors report the onset of arrhythmia during the inspiratory phase of the manoeuvre [3].

The large positive intra-thoracic pressures we observed during forced expiration may contribute to chronic deleterious consequences in susceptible individuals. Spirometry has been proposed to induce gastro-oesophageal reflux in approximately half of individuals referred for outpatient gastro-oesophageal reflux assessment [4]. While the exact mechanism of spirometry-induced gastro-oesophageal reflex is unknown, it is likely attributable to an increased intra-abdominal pressure, resulting in upward vectorial forces on gastric contents. Moreover, during activities that increase intra-abdominal pressure (e.g., deep inspiration, forced expiration, trunk flexion), the right crus of the diaphragm contracts to increase pressure on the lower oesophageal sphincter, thereby preventing gastric-oesophageal reflux [10]. As such, it is possible that reflux during forced expiration may be symptomatic of diaphragm weakness.

## Limitations

There are two limitations that should be considered when interpreting the data presented in this study. First, data were collected in a healthy cohort; i.e., participants free from cardiorespiratory disease, and the intra-thoracic pressures exhibited may not be representative of a clinical population. Further studies are needed to elucidate the typical response in, for example, chronic obstructive pulmonary disease (COPD) and asthma. Second, we recorded intra-thoracic pressures using oesophageal balloon-tipped catheters. While balloon catheters are widely used and exhibit excellent reliability, other common techniques involve multi-pair oesophageal electrode catheters, or pneumotachographs for the measurement of mouth-pressure. There is a lack of consistency in the literature with respect to the technique used; consequently, we urge caution when comparing among studies.

To conclude, this is the first report to characterise the intra-thoracic pressure-response to spirometry. We observed near maximal oesophageal pressures during expiration, and large peak-to-peak oesophageal pressure swings during an FVC manoeuvre which may part-explain some of the deleterious effects of pulmonary function testing. Future studies should aim to clarify causation, and comment on the mechanistic basis.

## Abbreviations

FVC: forced vital capacity
TLC: total lung capacity
RV: residual volume
FEV_1_: forced expiratory volume in one second
Pes: oesophageal pressure
ΔPes: oesophageal pressure swing (peak-to-peak) recorded during FVC manoeuvre
Pes_insp_: peak oesophageal pressure recorded during inspiratory phase of FVC
Pes_exp_: peak oesophageal pressure recorded during expiratory phase of FVC
P_Imax_: maximum static inspiratory oesophageal pressure
P_Emax_: maximum static expiratory oesophageal pressure
COPD: chronic obstructive pulmonary disease
